# Nucleases: From Primitive Immune Defenders to Modern Biotechnology Tools

**DOI:** 10.1111/imm.13884

**Published:** 2024-12-16

**Authors:** Frank J. Hernandez

**Affiliations:** ^1^ Department of Physics, Chemistry and Biology Linköping University Linköping Sweden; ^2^ Department of Bioengineering and Biosciences, TECNUN Navarra University Donostia Spain; ^3^ IKERBASQUE, Basque Foundation for Science Bilbao Spain

**Keywords:** autoinflammatory disease, immune homeostasis, inflammation

## Abstract

The story of nucleases begins on the ancient battlefields of early Earth, where simple bacteria fought to survive against viral invaders. Nucleases are enzymes that degrade nucleic acids, with restriction endonucleases emerging as some of the earliest defenders, cutting foreign DNA to protect their bacteria hosts. However, bacteria sought more than just defence. They evolved the CRISPR‐Cas system, an adaptive immune mechanism capable of remembering past invaders. The now‐famous Cas9 nuclease, a key player in this system, has been harnessed for genome editing, revolutionising biotechnology. Over time, nucleases evolved from basic viral defence tools into complex regulators of immune function in higher organisms. In humans, DNases and RNases maintain immune balance by clearing cellular debris, preventing autoimmunity, and defending against pathogens. These enzymes have transformed from simple bacterial defenders to critical players in both human immunity and biotechnology. This review explores the evolutionary history of nucleases and their vital roles as protectors in the story of life's defence mechanisms.

## Graphical abstract

The evolution of nucleases is a remarkable journey, starting as primitive bacterial defenders against bacteriophages. Over time, nucleases have adapted to specialised roles within the immune systems of all forms of life, culminating in humans, where DNases and RNases serve as key components of the immune system.

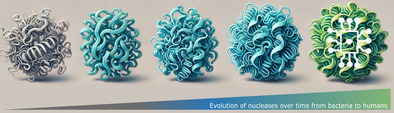

## 1 Introduction

The evolution of immune defence and bacteria's fight against invaders starts with the crucial ability to distinguish between friend and foe, self and non‐self [1, 2]. At the heart of this story are nucleases [3], those intriguing enzymes that have the power to degrade nucleic acids [4, 5]. From the simplest bacteria to the complexity of human beings, nucleases have quietly safeguarded life [6]. Their job is ancient and simple: destroy foreign DNA or RNA before it causes harm [7]. Yet, as life evolved, so did nucleases. What began as a bacterial weapon against viruses has now become a sophisticated system of immune regulation in higher organisms [4]. The earliest organisms, such as bacteria, had to confront bacteriophages, viruses that inject foreign DNA into the bacterial genome, hijacking the cell's machinery [8]. To overcome these attacks, bacteria evolved nucleases like restriction endonucleases, which recognise specific sequences in foreign DNA and cut them apart [9]. Fast forward through millions of years of evolution, and we see the same principles at play in all forms of life, including humans [10]. Nucleases now not only fend off viral invaders but also clean up after cell death, prevent excessive inflammation and regulate the immune system's balance [4]. This review traces the evolutionary journey of nucleases, starting from their humble origins in bacteria and progressing through their growing adaptability in more complex organisms. Over time, nucleases have evolved to protect life in diverse ways, adapting their functions to meet the challenges of different biological systems. They now play diverse and vital roles, with their ability to degrade nucleic acids becoming one of the most fundamental mechanisms of immune defence.

## 2 Bacterial Restriction Endonucleases: The Primitive Origin of Immune Defence

Imagine a time, billions of years ago, when bacteria waged war with bacteriophages. Amid these microscopic battles, nucleases emerged as bacterial defenders, equipped with precise tools capable of cleaving invading DNA. These defenders were known as restriction endonucleases [11], and they offered one of the earliest forms of immune protection [12]. They were simple but effective, spotting the invader's DNA and cutting it into useless fragments [13].

Bacteria produced different types of restriction endonucleases, each with its own strategy for combating foreign DNA:

- **Type I** endonucleases were a bit reckless, cutting DNA at random points far from the recognition site [14].
- **Type II** endonucleases were the precise surgeons, cutting DNA exactly at the recognition site, making them particularly useful in biotechnology [11].
- **Type III and IV** endonucleases exhibit unique characteristics in their DNA cleavage mechanisms [15, 16]. Type III restriction enzymes, like EcoP15I, recognise specific DNA sequences and cleave DNA at sites a short distance away, requiring ATP and cofactors for their activity [17]. In contrast, Type IV enzymes, such as McrBC, target modified DNA, particularly methylated or hydroxymethylated sequences, and typically degrade foreign DNA that bears these modifications [18]. These distinctions between Type III and IV enzymes allow them to play specific roles in DNA modification and degradation.

These nucleases protected bacteria by neutralising the viral invaders before they could replicate, setting the stage for the evolution of more complex immune mechanisms.

## 3 CRISPR‐Cas: The Adaptive Immunity of Bacteria

Bacteria, however, were not satisfied with mere defence; they developed memory. Thus, the CRISPR‐Cas system was born [19], a bacterial immune system with memory, allowing bacteria to recognise viruses they had encountered before [20]. CRISPR‐Cas acts like a high‐tech surveillance system, keeping a detailed record of viral DNA fragments in the bacterial genome [21]. When the same invader appears again, this ‘genetic blueprint’ allows CRISPR‐Cas to quickly recognise and neutralise the threat [22]. The most famous of these CRISPR‐associated nucleases is Cas9, which has revolutionised modern science by becoming a powerful tool for genome editing [23]. Cas9 works with a guide RNA that tells it exactly where to cut, creating breaks in the viral DNA and preventing the virus from replicating [23]. The human immune system, much like CRISPR systems in bacteria, employs memory cells such as B and T cells to ‘remember’ past infections. While more specialised and sophisticated, these cells retain the ancient concept of immune memory, allowing for rapid and targeted responses against recurring pathogens [24]. The CRISPR‐Cas system exemplifies the ingenuity of nature, showing how even the simplest organisms can evolve complex mechanisms for survival.

## 4 The Evolution of DNases and RNases From Archaea to Mammals

As life evolved to complex organisms, nucleases transformed from simple defenders into highly specialised enzymes crucial for survival. In archaea, nucleases evolved to be highly specialised for extreme environments, playing key roles in both defence against invaders and maintaining genomic stability under stress [25]. For instance, Holliday junction endonuclease Hjc from *Sulfolobus solfataricus* resolves recombination junctions in sulphur‐rich, acidic springs [26]. These enzymes underscore the resilience and functionality of archaeal nucleases in extreme environments. In marine cyanobacteria like *Synechococcus* and *Prochlorococcus*, several types of nucleases associated with defence systems have been reported. For example, Type I restriction‐modification (R‐M) systems have been found in *Synechococcus* strains such as RS9917 and WH5701, while potential Type III R‐M systems are present in *Prochlorococcus* strains MED4 and MIT9312. This showcases the diversity and variability of nuclease‐based systems developed across different forms of life [27]. In protists such as *Dictyostelium discoideum*, DNase II helps clear cellular debris during autophagy and apoptosis, preventing immune activation by degrading DNA from dead cells. This genomic cleanup function acts as a precursor to immune regulation in higher organisms [28].

In yeast such as *Saccharomyces cerevisiae* , nucleases such as Exo1 are involved in DNA mismatch repair, protecting the genome from errors during replication [29]. Although fungi do not have immune systems, these mechanisms are critical for maintaining genetic integrity [30]. In invertebrates like the nematode *Caenorhabditis elegans* , CPS‐6 is an apoptotic nuclease that prevents harmful immune activation, reflecting early immune‐like regulatory roles [31]. In flatworms such as *Dugesia japonica* , DNase II has been reported as an important component of the antimicrobial response, contributing to immune regulation and the survival of this organism [32].

In molluscs like *Mytilus galloprovincialis* , nucleases such as RNase T2 are involved in regulating immune responses, with overexpression during infection. RNase T2 acts as a chemokine, attracting immunocytes to infected areas and assisting with the inflammatory response [33]. Similarly, in insects like *Drosophila melanogaster* , DNase II ensures the proper clearance of apoptotic DNA, preventing overactive immune responses, which is essential for maintaining proper immune function in *Drosophila* [34]. In vertebrates such as zebrafish ( *Danio rerio* ), DNase II functions as part of the innate immune system, degrading DNA from dying cells and preventing autoimmune responses [35]. In amphibians like *Xenopus laevis* , DNase I (DNase γ) helps process apoptotic DNA, playing a critical role in nucleosomal DNA fragmentation during apoptosis, marking a key evolutionary step where nucleases contribute to immune defence [36].

In birds like chickens ( *Gallus gallus* ), RNase L plays an important role in the antiviral response by degrading viral RNA during infections, highlighting how nucleases became integrated into vertebrate immune systems [37]. Finally, in mammals, DNase I and RNase A family are vital in maintaining immune balance by degrading self‐DNA and viral RNA, preventing autoimmune responses and regulating inflammation. These nucleases represent the sophisticated roles they have acquired in managing immune responses in higher mammals [38].

Overall, the evolutionary journey of nucleases, from early prokaryotes to mammals, illustrates how these enzymes transitioned from basic genome maintenance roles to specialised immune regulatory functions. In early organisms such as bacteria, archaea and fungi, nucleases primarily defended the genome. As multicellular organisms evolved, nucleases took on more immune‐specific roles, such as clearing apoptotic cells and combating pathogens. In vertebrates and higher organisms, nucleases became integral to immune system balance, preventing autoimmunity and defending against viral infections.

## 5 Nucleases Involve in the Human Immune Response

The evolution of nucleases in humans reflects the growing complexity of the immune system as organisms faced increasingly diverse threats from pathogens. Over time, nucleases have specialised to fulfil distinct roles in maintaining immune homeostasis. DNases and RNases have evolved to play key roles in the immune response, helping to clear pathogenic DNA and RNA, regulate inflammation and prevent autoimmune reactions. The most representative nucleases in human immunity are presented below.

### 5.1 DNases in Human Immunity

DNases are essential for degrading extracellular DNA, which can accumulate during infections, inflammation and cell death. Without proper degradation, this DNA can trigger immune responses that lead to chronic inflammation and autoimmunity. The human genome contains four DNase I and two DNase II genes, along with TREX nucleases and DNase IL‐3 [39]. Importantly, the substrate specificity for DNase I and DNase II is influenced by factors such as pH, with optimal catalytic activity ranging from 6.5 to 8.0 for DNase I and 4.8 to 5.2 for DNase II. Additionally, their cleavage patterns differ: DNase I cleaves DNA to produce oligonucleotides with 5΄‐phosphate and 3΄‐hydroxyl ends, while DNase II generates oligonucleotides with 5΄‐hydroxyl and 3΄‐phosphate ends [40]. A short description of each type of DNase is provided as follows:

- **DNase I**: The most well‐known human DNase, DNase I, degrades extracellular DNA from apoptotic cells, pathogens, and neutrophil extracellular traps (NETs) [41]. By cleaving DNA before it can be detected by pattern recognition receptors like TLR9, DNase I helps to prevent inappropriate immune activation [42]. DNase I deficiency is associated with the development of systemic lupus erythematosus (SLE), where self‐DNA accumulates and triggers autoimmunity [43].
- **DNase II**: This lysosomal enzyme is responsible for degrading DNA within phagosomes. It plays a key role in clearing the DNA of engulfed pathogens or apoptotic cells. DNase II is critical for maintaining immune tolerance by ensuring that self‐DNA is efficiently degraded [44]. Deficiency in DNase II can lead to the accumulation of undegraded DNA, resulting in chronic inflammation and autoimmune conditions [45].
- **TREX1**: This cytoplasmic exonuclease is essential for degrading aberrant DNA, including that generated during replication stress and viral infection [46]. TREX1 prevents the accumulation of cytosolic DNA, which can activate the cGAS‐Stimulator of Interferon Genes (STING) pathway and trigger type I interferon production [47]. Mutations in TREX1 are associated with autoimmune diseases such as Aicardi‐Goutières syndrome [48] and familial chilblain lupus [49].
- **TREX2**: This exonuclease is crucial for genome integrity in skin cells, is a homologue of TREX1 but plays a distinct role by linking transcription and mRNA export in mammalian cells.
- **DNase γ (DNase IL‐3)**: DNase γ is involved in apoptotic DNA degradation and plays a role in DNA fragmentation during the later stages of apoptosis [50]. It acts downstream of caspase activation, contributing to the clearance of nuclear DNA from cells undergoing programmed death [50]. By ensuring that DNA is fragmented and cleared, DNase γ prevents immune activation by apoptotic debris [51].

### 5.2 RNases in Human Immunity

RNases are equally important for maintaining immune homeostasis, particularly in degrading viral RNA and regulating inflammatory responses [52, 53].

- **RNase 1**: The most abundant RNase in human blood plasma, RNase 1 degrades extracellular RNA, which can act as a damage‐associated molecular pattern (DAMP). By breaking down extracellular RNA, RNase 1 prevents inappropriate immune activation and reduces inflammation [54].
- **RNase 2 (eosinophil‐derived neurotoxin, EDN)**: RNase 2 is secreted by eosinophils and has antiviral activity, particularly against single‐stranded RNA viruses such as respiratory syncytial virus (RSV). Additionally, RNase 2 degrades inflammatory RNA molecules, helping to modulate immune responses during infection [54].
- **RNase 3 (eosinophil cationic protein, ECP)**: Like RNase 2, RNase 3 is secreted by eosinophils and exhibits antimicrobial and antiviral properties. It is especially effective against parasites, making it an important factor in the immune response to parasitic infections [55].
- **RNase 4**: RNase 4 degrades extracellular RNA and helps maintain homeostasis by preventing the accumulation of RNA outside of cells. It may also play a role in modulating immune responses during tissue injury and inflammation [56].
- **RNase 5 (angiogenin)**: RNase 5, or angiogenin, has dual roles in immune and nonimmune functions. In addition to promoting angiogenesis (the formation of new blood vessels), RNase 5 has antimicrobial properties and can degrade viral RNA, contributing to immune defence against infections [54].
- **RNase 6**: RNase 6 is involved in antimicrobial defence, particularly against bacteria. It is secreted by neutrophils and helps eliminate bacterial RNA, supporting the immune system's efforts to combat bacterial infections [57, 58].
- **RNase 7**: Found in epithelial tissues, RNase 7 has potent antimicrobial activity against bacteria, fungi and viruses. By degrading microbial RNA, RNase 7 helps prevent the spread of infections on mucosal surfaces and in tissues like skin [58, 59].
- **RNase 8**: RNase 8 is primarily expressed in the placenta and has a more restricted tissue distribution compared to other members of the RNase A superfamily. Although its precise role in immunity is not as well understood, RNase 8 is believed to contribute to host defence, particularly in the context of pregnancy [54].
- **RNase L**: RNase L is a key player in the antiviral response. It is activated by the OAS (oligoadenylate synthetase) pathway in response to interferon signalling. Upon activation, RNase L degrades viral and host RNA, inhibiting viral replication. Its activity is a crucial component of the innate immune response to RNA viruses [60].
- **RNase T2**: RNase T2 is an evolutionarily conserved enzyme with broad roles in RNA degradation and immune regulation. It is involved in the degradation of both endogenous and exogenous RNA, helping to control inflammation and maintain immune homeostasis [61, 62]. Additionally, RNase T2 may contribute to autophagy processes, where it facilitates the degradation of RNA within lysosomes, further supporting cellular cleanup and protection against immune overreaction [63].

Collectively, these RNases form a critical part of the human immune system, providing defence against a wide range of pathogens, regulating immune responses and helping to prevent excessive inflammation [64].

## 6 Nuclease Activity and Oligonucleotides as Immune Activators

Oligonucleotides, the short fragments of nucleic acids produced by nucleases during the degradation of DNA and RNA, play an essential role as signalling molecules in the immune system [65, 66]. This process begins when nucleases target foreign DNA or RNA from pathogens or damaged host cells. The resulting oligonucleotide fragments are not merely by‐products of degradation but act as critical danger signals, alerting the immune system to potential threats [67]. Through a variety of pathways, these small fragments can activate immune cells, triggering inflammation and antiviral responses and ensuring a rapid and coordinated defence [68]. The detection of foreign nucleic acids, such as microbial or pathogenic DNA, depends on several factors, including their availability, localisation and structural characteristics [2]. Nucleases such as DNase IL‐3 [69] and DNase I play a crucial role in degrading nucleic acids before they can be recognised by immune receptors [2]. Apoptotic DNA fragmentation is mediated by CAD (caspase‐activated DNase), while DNA digestion in macrophage lysosomes is handled by DNase II [70]. Double‐stranded DNA (dsDNA) fragments can activate the immune system in a sequence‐independent manner, even with fragments as short as 25 base pairs (25mer), typically when introduced into the cytoplasm due to infection or cell damage [71, 72]. This activation induces the expression of MHC molecules and other critical genes for antigen presentation [72]. Recognition of dsDNA by cGAS is also sequence‐independent, with cGAS favouring B‐form DNA and showing a preference for longer DNA fragments, which tend to activate cGAS more efficiently [73]. cGAS responds to mitochondrial or genomic DNA leakage caused by stress, as well as detecting dsDNA, ssDNA and RNA–DNA hybrids from pathogens [74, 75]. In contrast, RIG‐I primarily detects short double‐stranded RNAs (20–40 bp), while MDA5 requires much longer dsRNAs, over 1000 base pairs, for effective signalling. This length‐dependent recognition is vital for distinguishing self from foreign nucleic acids [73].

One of the key pathways through which oligonucleotides signal the immune system is via Toll‐Like Receptor 9 (TLR9). TLR9 is located within the endosomes of immune cells such as dendritic cells and macrophages, where it recognises unmethylated CpG motifs, which are common in bacterial and viral DNA [42]. When nucleases digest pathogenic DNA, they produce oligonucleotides rich in these motifs [42]. Upon recognition by TLR9, the immune system is prompted to release proinflammatory cytokines and type I interferons, creating a robust immune response aimed at clearing the infection [76]. This pathway is critical in bacterial and viral infections, where the immune system must quickly differentiate between foreign nucleic acids and those belonging to the host [42].

Another significant mechanism is the cGAS‐STING pathway, which detects cytosolic double‐stranded DNA (dsDNA) oligonucleotides [77]. Normally, DNA is confined to the nucleus and mitochondria, so the presence of dsDNA in the cytoplasm is a signal of cellular stress or infection [77]. Nucleases generate these DNA fragments from either damaged cells or intracellular pathogens like viruses. The enzyme cyclic GMP‐AMP synthase (cGAS) binds to these DNA fragments, synthesising cyclic GMP‐AMP (cGAMP), which in turn activates the STING receptor [78]. This activation leads to the production of type I interferons and other inflammatory molecules that promote antiviral defences [78]. The cGAS‐STING pathway thus plays a pivotal role in recognising both viral infections and cellular damage.

Beyond DNA, RNA fragments also serve as critical signalling molecules. The RIG‐I and MDA5 pathways are part of the body's defence against viral infections, where short RNA oligonucleotides generated from viral genomes are recognised [79]. It is worth noting that RNase L has been postulated as one of the RNases that produce small self‐RNAs from cellular RNA, inducing IFN‐beta expression and signalling involving RIG‐I and MDA5 [80]. RIG‐I detects short 5′‐triphosphate RNA fragments, while MDA5 responds to long double‐stranded RNA (dsRNA) [79]. These receptors trigger downstream signalling cascades that induce the production of type I interferons and proinflammatory cytokines, enhancing the immune system's ability to halt viral replication and eliminate infected cells [79]. This RNA sensing mechanism underscores the versatility of oligonucleotides as immune activators, whether the nucleic acid fragments are from DNA or RNA [81].

In summary, oligonucleotides serve as powerful activators of the immune system through multiple pathways. By recognising these small nucleic acid fragments, the immune system is able to detect infections, cellular damage and stress, mounting appropriate responses that range from inflammation to the production of antiviral molecules [82]. The ability of oligonucleotides to function as signalling molecules highlights the sophistication of immune surveillance, which relies on the detection of molecular patterns that signal danger, whether from external pathogens or internal tissue damage. This process is crucial not only for innate immunity but also for maintaining tissue homeostasis and promoting repair after injury.

## 7 Biotechnology Approaches Based on Nucleases

Nucleases, the enzymes responsible for degrading nucleic acids such as DNA and RNA, play a critical role in biotechnology and biomedical research due to their remarkable precision and efficacy [83]. These enzymes are essential in all forms of life, contributing to processes such as immune defence and cellular maintenance [84]. However, their utility extends far beyond natural functions. In modern biotechnology, nucleases are harnessed as powerful tools for cutting, modifying and editing genetic material, driving innovations in fields such as genetic engineering, therapeutic development and industrial applications [85].

DNA at precise sequences, enabling gene splicing and cloning [86]. This breakthrough facilitated advancements such as the creation of genetically modified organisms (GMOs) [87], the production of biopharmaceuticals and gene therapies [88]. Following this, zinc finger nucleases (ZFNs) were introduced, allowing for more precise DNA modifications by targeting specific sequences, opening new possibilities for gene editing with greater accuracy [89]. Transcription activator‐like effector nucleases (TALENs) further refined gene‐editing capabilities, enabling scientists to make specific changes to DNA with even more precision for research and therapeutic purposes [90]. The most recent advancement, CRISPR‐Cas9, revolutionised genetic engineering by providing highly targeted genome editing tools, making it a key player in correcting genetic mutations and advancing treatments for diseases [91]. Beyond their role in genetic engineering, nucleases have significant therapeutic applications. CRISPR‐Cas9 enables the precise editing of defective genes, offering potential cures for conditions such as cystic fibrosis [92] and sickle cell anaemia [93]. Nucleases are also being explored as therapeutic agents in oncology, targeting cancer cells to inhibit tumour growth [94], and play a crucial role in antiviral therapies by degrading viral genomes, providing methods to combat persistent infections such as HIV [95] and hepatitis [96].

Looking to the future, nuclease‐based therapies hold great promise, especially in the field of personalised medicine. One emerging approach is the use of nucleases as biomarkers and therapeutic activators in systems such as Therapeutic Oligonucleotides Activated by Nucleases (TOUCAN) [97]. This concept involves designing prodrug systems that remain inactive until they encounter nucleases associated with a specific disease or infection, allowing for precise, localised treatment. This strategy could revolutionise targeted therapies for bacterial infections, cancers, and autoimmune diseases, further expanding the therapeutic utility of nucleases.

Nucleases have also become valuable tools in molecular diagnostics, particularly in techniques such as CRISPR‐based diagnostic platforms, such as SHERLOCK [98] and DETECTR [99], utilise nucleases such as Cas12 and Cas13 to detect specific genetic sequences, offering rapid and highly sensitive diagnostics for pathogens, genetic mutations and other molecular targets [100].

## 8 Conclusion

The evolutionary journey of nucleases, from simple bacterial defence mechanisms to complex regulators of human immunity, reveals their remarkable adaptability. These enzymes, with their ancient roots, continue to shape the way organisms defend against invaders, regulate immune responses and maintain balance within the body.

Their ability to generate oligonucleotide danger signals underscores their role as both protectors and sentinels, bridging the gap between innate and adaptive immunity. As research into nucleases continues, these enzymes may offer new avenues for treating autoimmune diseases, chronic inflammation and even infections, highlighting their enduring relevance in health and disease.

Nucleases also hold the potential to revolutionise modern medicine. The CRISPR‐Cas system has been harnessed in modern biotechnology for gene editing [21]. By programming Cas9 to target specific DNA sequences, scientists can induce precise cuts at desired locations in the genome [101]. This technology has opened new doors in medicine, allowing for potential treatments for genetic disorders by editing defective genes. Another recent approach utilising nucleases as biomarkers is known as TOUCAN [97]. This prodrug system employs chemically modified oligonucleotide substrates as a drug delivery mechanism, designed to specifically target the site of bacterial infections. The TOUCAN system remains inactive until it encounters nucleases associated with the bacterial infection, at which point it degrades, releasing the therapeutic drug directly at the infection site [97]. This innovative strategy paves the way for developing personalised antibiotics and could potentially be adapted for other conditions, such as immune system activation and cancers, where nucleases serve as biomarkers [102].

In the story of life, nucleases are guardians of genomic integrity, masters of immune regulation, and innovators in both natural evolution and biotechnology. Evolving over millions of years, they began as primitive defence mechanisms in bacteria, cutting foreign DNA and protecting early life. As organisms became more complex, nucleases have shown remarkable adaptability, becoming integral to the evolution of all forms of life by maintaining genome stability, repairing DNA and regulating immune responses through the recognition of foreign genetic material and the triggering of immune defences. Their journey from ancient defence systems to modern biotech tools underscores their enduring importance in health, disease and the future of medicine.

## Conflicts of Interest

The author declares no conflicts of interest.

## Data Availability

The data that support the findings of this study are available from the corresponding author upon reasonable request.
